# Influenza virus causes lung immunopathology through down-regulating PPARγ activity in macrophages

**DOI:** 10.3389/fimmu.2022.958801

**Published:** 2022-08-25

**Authors:** Hongbo Zhang, Taylor Alford, Shuangquan Liu, Dongming Zhou, Jieru Wang

**Affiliations:** ^1^ Department of Pediatrics, University of Pittsburgh School of Medicine, Pittsburgh, PA, United States; ^2^ Department of Medicine, National Jewish Health, Denver, CO, United States; ^3^ Department of Clinical Laboratory, The First Affiliated Hospital of University of Southern China, Hengyang, Hunan, China; ^4^ Department of Pathogen Biology, School of Basic Medical Sciences, Tianjin Medical University, Tianjin, China

**Keywords:** lung macrophage, PPARγ, influenza, PPARγ agonist, acute lung injury

## Abstract

Fatal influenza (flu) virus infection often activates excessive inflammatory signals, leading to multi-organ failure and death, also referred to as cytokine storm. PPARγ (Peroxisome proliferator-activated receptor gamma) agonists are well-known candidates for cytokine storm modulation. The present study identified that influenza infection reduced PPARγ expression and decreased PPARγ transcription activity in human alveolar macrophages (AMs) from different donors. Treatment with PPARγ agonist Troglitazone ameliorated virus-induced proinflammatory cytokine secretion but did not interfere with the IFN-induced antiviral pathway in human AMs. In contrast, PPARγ antagonist and knockdown of PPARγ in human AMs further enhanced virus-stimulated proinflammatory response. In a mouse model of influenza infection, flu virus dose-dependently reduced PPARγ transcriptional activity and decreased expression of PPARγ. Moreover, PPARγ agonist troglitazone significantly reduced high doses of influenza infection-induced lung pathology. In addition, flu infection reduced PPARγ expression in all mouse macrophages, including AMs, interstitial macrophages, and bone-marrow-derived macrophages but not in alveolar epithelial cells. Our results indicate that the influenza virus specifically targets the PPARγ pathway in macrophages to cause acute injury to the lung.

## Introduction

Influenza A virus (IAV) is a common pathogen causing respiratory illness. Pathology is determined by pathogens and the host (1). The easy-mutation nature of the influenza virus made it a challenging to develop a universal vaccine to protect the susceptible population (2, 3). Therefore, identifying the host-derived mechanism for influenza-associated diseases is critical for developing an effective prevention or treatment strategy for combating influenza infection.

Peroxisome proliferator-activated receptor gamma (PPARγ*)* is a member of the nuclear hormone receptor superfamily of ligand-activated transcription factors that regulate the expression of genes involved in reproduction, metabolism, development, and immune responses (4, 5). PPARγ is a well-known anti-inflammatory transcription factor (6, 7). It has been reported that respiratory virus, such as a respiratory syncytial virus (RSV), alter PPARγ expression (6, 7). In addition, PPARγ ligands are proposed as anti-SARS-CoV-2 drugs based on their anti-inflammatory, antioxidant and immunomodulatory properties (8, 9). Several PPARγ agonists have been documented to attenuate inflammation and alleviate influenza infection in mouse studies. Cloutier et al. found that 15-Deoxy-Delta-12,14-prostaglandin J2 (15d-PGJ2), a ligand of PPARγ, protects mice against lethal influenza infection (10). Several reports suggest that IAV infection downregulates PPAR-γ expression in mouse macrophages (11, 12). However, it is not known whether the influenza A virus directly hijacks PPARγ signaling in human primary alveolar macrophages.

PPARγ is predominantly expressed in alveolar macrophages (AMs) (13). Alveolar macrophages are the lung’s immune effector and play a central role in maintaining lung homeostasis by repairing tissue damage and phagocytosis of invading pathogens (14). During infection, AMs release a lot of proinflammatory cytokines to recruit immune cells to the infection site (13). However, the excessive proinflammatory response, the so-called cytokine storm, contributes significantly to the tissue damage (15). Our previous study with genome profiling of human AMs in response to influenza infection suggests that influenza may reduce PPARγ gene expression (16). Therefore, we hypothesize that the influenza A virus surpresses the PPARγ pathway, therefore contributes to the dysregulation of the host immune response.

The present study first examined whether influenza infection impaired PPARγ signaling *in vitro* in human primary lung cells, including the alveolar epithelial and macrophage cells. We then addressed whether activation of PPARγ by a commercial agonist would reduce the proinflammatory response in human AMs *in vitro* and protect infected mice from severe lung injury and inflammation *in vivo.* We demonstrated that influenza infection impaired PPARγ signaling, which led to an excessive proinflammatory response in human AMs but not in epithelial cells and lung inflammation and pathology *in vivo*. Our results revealed a novel mechanism of influenza-induced acute lung injury.

## Materials and methods

### Influenza infection of human AMs

Influenza H1N1 virus A/PR8/Puerto Rico/34 (PR8) and 2009 H1N1 pandemic virus A/California/04 (CA04) were prepared as described previously (17–19). According to the standard procedure, the contemporary H3N2 virus vz18B2 was created by reverse genetics directly from a human swab specimen collected in New York State a human swab collected in New York state and kindly provided by Dr. Wentworth (20). Human AMs were isolated from unidentified donor lungs, which were not suitable for transplantation, and donated for medical studies as described previously (18, 19). Cultured AMs were inoculated with the influenza virus at a multiplicity of infection (moi) of 1 for 1 h. Cells were harvested at 24 h post-inoculation (hpi). The Committee for the Protection of Human Subjects at National Jewish Health and University of Pittsburgh Committee for Oversight of Research and Clinical Training involving Decedents (CORID) have approved this study.

### Mouse influenza infection

Eight to ten-week-old C57BL/6 mice were purchased from The Jackson Laboratory (Bar Harbor, Maine). Mice were maintained under specific pathogen-free conditions within the animal facility at the Children**’**s Hospital of Pittsburgh of University of Pittsburgh Medical Center. Animal studies were conducted with approval from the University of Pittsburgh Institutional Animal Care and Use Committee. For influenza infection, mice were intranasally challenged with 1000 pfu of PR8 virus diluted in 50 μl of sterile PBS or 50 μl of PBS control. Following infection, mice were monitored daily for weight loss and signs of clinical illness and 3 or 5 mice were sacrificed 3 days after the virus challenge.

### Mouse bronchoalveolar lavage and lung tissue processing

At the indicated time, mice were euthanized by intraperitoneal injection of a leathal dose of sodium pentobarbital. The whole-lungs lavage was performed with 1 ml sterile saline solution, and bronchoalveolar lavage fluid (BALF) was collected and centrifuged at 4°C, 3000 rpm for 10 min. An aliquot of 100ul cell-free BALF was snap-frozen by the dry ice-ethanol bath to evaluate viral burden by standard Plaque Assay as previously described (18, 19). The remaining BALF was stored at -80°C for detection of albumin, lactate dehydrogenase (LDH), and cytokine by ELISA. BAL cell cytospin slides were stained with a HEMA-3 stain kit (Fisher Scientific, Waltham, MA) for inflammatory cell differential counts. For histology staining, the right superior lobe was fixed in 10% neutral buffered formalin. The right lobe was saved at -80°C for protein extraction. The remaining lung lobes were collected and homogenized in 1 ml of sterile ice-cold PBS at 4°C using a gentle MACS Dissociator (Miltenyi Biotech, San Diego, CA). The post-caval lobe was saved at -80°C for RNA extraction.

### Treatment with PPARγ agonist and antagonist

For a PPARγ agonist and antagonist experiment in human AMs, troglitazone (5 μM) (Sigma, St Louis, MO) was given 1 h before or right after infection. PPARγ antagonist T0070907 or GW9662 (Sigma, St Louis, MO) were given 1h before infection. After infection, cells were cultured with agonists or antagonists for another 24 h.

For treatment with mice, 10 mg/kg troglitazone was injected intraperitoneally prior to flu infection and daily after infection for 5 days. Mice were observed daily for daily activity and weight loss. On Day 5 post flu inoculation, mice were sacrificed. 2 mg/kg T0070907 or GW9662 was injected intraperitoneally 1 day prior to flu infection and daily after infection for 2 days. Mice were observed daily for daily activity, weight loss, and the mice were sacrificed on Day 3 post flu infection.

### PPARγ transcription activity assay

The nuclear protein from control and virus-infected human AMs and mouse lungs was extracted using Pierce nuclear/cytoplasmic protein extraction kit (ThermoFisher Scientific, Waltham, MA). Following the manufacturer’s instruction, an equal amount of nuclear protein from control and infected samples was used to evaluate the PPARγ activity using the TransAM PPARγ kit (Active Motif Inc., Rixensart, Belgium). The kit includes a 96-well plate coated with with an immobilized oligonucleotide containing the PPARγ response element (5’-AACTAGGTCAAAGGTCA-3’) for measuring PPARγ activity. The active form of PPARγ contained in nuclear extract specifically binds to the oligonucleotide. The primary antibodies used in the TransAM PPARγ kit recognize an accessible epitope on PPARγ protein upon DNA binding. A horseradish peroxidase-conjugated secondary antibody is used for the spectrophotometric quantification.

### Real-time RT-PCR

Total RNA was extracted from human and mouse cells using the RNeasy kit (QIAGEN S.A., Courtaboeuf, France). RNA purity and integrity were examined by Nanodrop spectrophotometric analysis. Following the manufacturer’s instructions, 1 μg of total RNA was reverse-transcribed into cDNA using the qScript™ cDNA Synthesis kit (Quanta Bioscience, Gaithersburg, MD). cDNA was then used in standard real-time PCR to measure gene expression using the Applied Biosystems 7900HT. Reaction conditions were 95°C for 15 s and 60°C for 1min, repeated for 40 cycles, with a 10 min hot start at 95°C. Relative mRNA level was quantified using the 2^ΔCt^ method and standardized by GAPDH. All TaqMan real-time PCR probes were purchased from Life Technologies (Carlsbad, CA). They are human PPARγ (Hs00234592_m1), human IL-1B (Hs00174097_m1), human GAPDH (Hs02758991_g1), mouse PPARγ (Mm00440940_m1), mouse Mx1 (Mm00487796_m1), and mouse GAPDH (Mm99999915_g1).

### Statistical analysis

All data analyses were performed with Prism 9.3.0 (GraphPad, La Jolla, CA). Mann–Whitney U test was used to compare gene expression and viral replication between two groups. The two-tailed Student *t*-test was used for the comparisons between the two groups. A one-way ANOVA test was used for the comparison of the three groups.

## Results

### Influenza infection impairs the PPARγ pathway in human primary AMs

**Figure 1:**
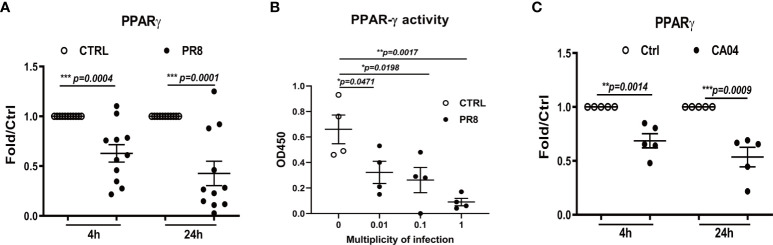
Influenza infection reduces PPARG gene expression and transcription activity in human alveolar macrophages(AMs). Cultured human AMs were infected with PR8 virus **(A, B)** or CA04 virus **(C)** at MOI=1, at 4 or 24 hpi, the cell pellet was harvested for RNA extraction, and the mRNA level of PPARγ was determined by RT-qPCR **(A, C)**. Human AM was infected with PR8 at MOI=0.01, 0.1, or 1, harvested the cell pellet for the nuclear protein extraction at 24 hpi, and the PPARγ transcriptional activity of PPARγ was determined **(B)**. Unpaired t test was applied for the statistical analysis of **(A, C)** and Ordinary one-way ANOVA was applied for B. *p < 0.05, **p < 0.01, and ***p < 0.001.

To confirm whether influenza A infection impairs PPARγ anti-inflammatory pathway in human primary AMs, cultured cells were infected with H1N1 virus PR8, and gene expression and transcription activity of PPARγ was measured at 4 and 24 h post-infection (hpi). Our results suggested that PR8 infection decreased mRNA of PPARγ in human AMs from 10 tested donors at 4 hpi and a further reduction at 24 hpi (**Figure 1A**). Since PPARγ is a transcription factor, we extracted cellular nuclear protein from infected cells at 24 hpi and further determined the transcription activity of PPAR**γ** using the TransAM kit (Active motif) in cells isolated from 4 donors. The data in **Figure 1B** show a dose-dependent decrease in PPARγ activity and virus.

In addition, we infected human AMs with the 2009 pandemic (H1N1pdm09) virus CA04. As we previously reported, this virus caused a much lower infection than PR8 (19), but it still significantly reduced PPARγ expression (**Figure 1C**) at both 4 and 24 hpi. Our previous study found that PR8 infection reduced the expression of important scavenger receptors, including CD36, the direct target gene of PPARγ in human AMs (16). Altogether, these data indicate that H1N1 infection impairs the PPARγ signaling pathway in human AMs.

### PPARγ is critical for controlling influenza virus-induced proinflammatory response in human AMs.

**Figure 2:**
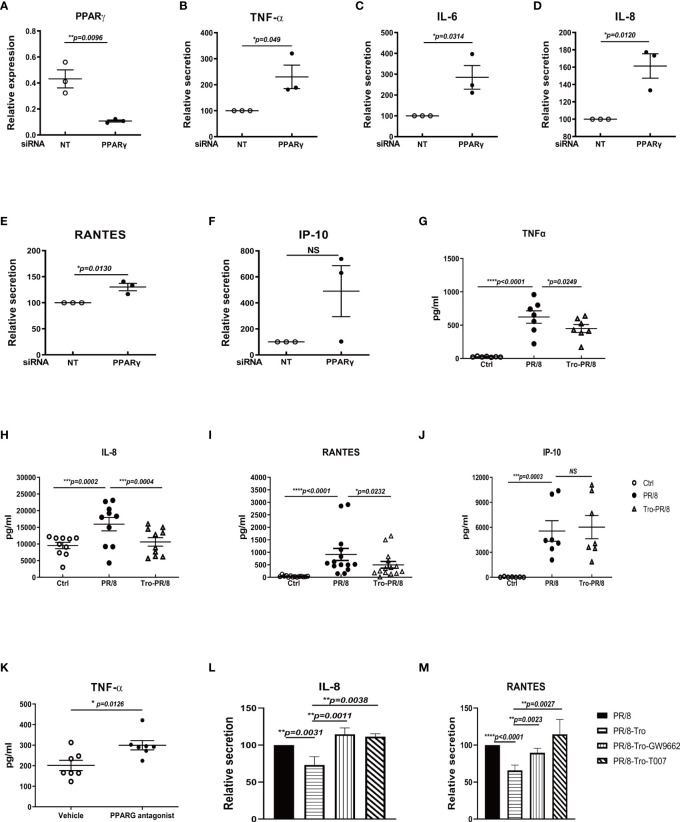
PPARγ is critical for controlling influenza virus-induced proinflammatory response in human AMs. Human AMs isolated from three donors were transfected with PPARγ specific siRNA or nontarget control siRNA(NT) 48 h before infections with PR8(MOI=1). At 24 h after infection, RNA**(A-F)** was extracted for examining PPARγ gene expression, and cell-free culture supernatants were collected to detect cytokines using DuoSet ELISA kits from R&D Systems. **(A)** PPARγ mRNA level. **(B)** TNFα. **(C)** IL-6. **(D)** IL-8. **(E)** RANTES. **(F)** IP-10. **(G–J)** For the experiment with PPARγ agonist, troglitazone (5 μM) (Sigma, St Louis, MO) in human AMs, was given right after infection. PPARγ antagonist T0070907 or GW9662 (Sigma) were given 1h before infection. At 24 h after infection, cell-free culture supernatants were collected to detect cytokines using DuoSet ELISA kits from R&D Systems. **(G)** TNFα. **(H)** IL-8. **(I)** RANTES. **(J)** IP-10. Isolated human AMs were treated with PPARγ agonist, troglitazone (5 μM) in human AMs right after PR/8 infection. PPARγ antagonist T0070907 or GW9662 (Sigma) were given 1h before infection. After infection, cells were treated with agonists or antagonists for another 24 h. At 24 h after infection, cell-free culture supernatants were collected to detect cytokines using DuoSet ELISA kits from R&D Systems. **(K)** TNFα. **(L)** IL-8. **(M)** RANTES. Unpaired t test was applied for comparison of two groups, while one-way ANOVA was used for comparison of more than two groups. ns means no significance. *p < 0.05, **p < 0.01, and ***p < 0.001.

We and others reported that the influenza A virus stimulated a predominant proinflammatory response in human AMs (16, 18, 19). To determine if this strong inflammatory response is due to dampened PPARγ signaling, we did several experiments to knockdown PPARγ using PPARγ-specific siRNA or alter PPARγ activity using a commercially available PPARγ agonist or antagonists prior to virus infection and measured proinflammatory cytokine secretion. PPARγ-specific siRNA treatment reduced around 90% of PPARγ expression (**Figure 2A**) and resulted in significantly upregulated secretion of TNF-α, IL-6, IL-8, and RANTES, but not IP-10 (**Figures 2B–F**). On the other hand, activated PPARγ by an agonist, Trog, significantly reduced virus-stimulated TNF- α, IL-8, and RANTES but not IP-10 (**Figures 2G-J**). Similar results were observed in contemporary H3N2 viral infection treated with PPARγ agonist (**Supplementary Figure 1**). On the contrary, PPARγ antagonist further enhanced virus-stimulated TNF-α (**Figure 2K**). As expected, the decreased secretion of IL-8 and RANTES caused by PPARγ agonist treatment was restored by subsequent antagonist treatment (**Figures 2L**, **M**). These results indicate that PPARγ is critical for keeping the proinflammatory response on check.

### PPARγ agonist does not alter influenza-induced IFN production as well as IFN downstream signaling

**Figure 3:**
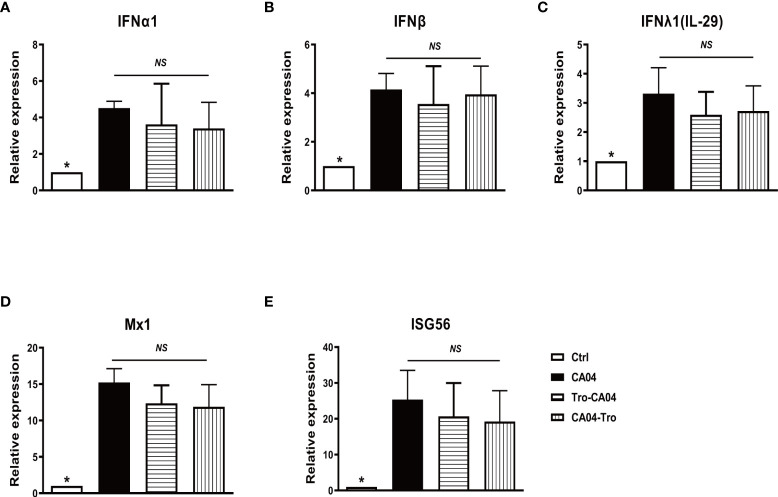
PPARγ agonist does not alter influenza-induced IFN production and IFN downstream signaling. Cultured human AMs were infected with CA04 at MOI=1, and PPARγ agonist troglitazone (5 μM) (Sigma, St Louis, MO) was given 1 h before or after infection. After infection, cells were cultured with an agonist for another 24 h. At 4 or 24 hpi, the cell pellet was harvested for RNA extraction, and the expression level of indicated genes was determined by RT-qPCR. **(A)** IFNα. **(B)** IFNβ. **(C)** IFNλ. **(D)** Mx1. **(E)** ISG56. Mann-Whitney test was apllied for the statistical analysis between CA04 and non- infected control conditions. Ordinary one-way ANOVA was applied for the statistical analysis among CA04, Tro-CA04 & CA04-Tro conditions. ns means no significance. *p < 0.05.

Production of IFN is a major antiviral defense of human AMs in response to influenza infection. To determine whether the administration of PPARγ agonist alters IFN response, we examined gene expression of interferons. Treatment of human AMs with PPARγ agonists did not alter PR8-induced mRNA production of IFN-α, IFN-β, and IFN-Λ1 (**Figures 3A–C**). PPARγ agonist also did not alter CA04-induced expression of IFN genes and antiviral genes Mx1 and ISG56 (**Figures 3D**, **E**). These results suggest that activation of PPARγ does not interfere with the host antiviral pathway.

### Influenza infection reduces mRNA and transcription activity of PPARγ in mice

**Figure 4:**
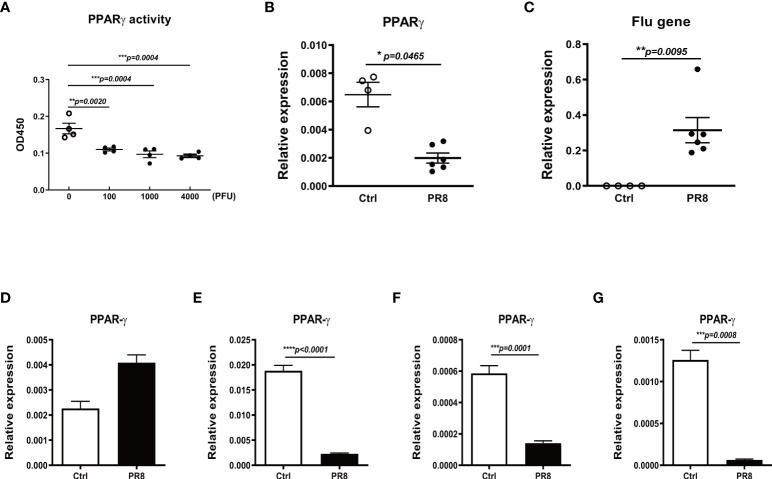
Influenza infection reduces PPARγ expression and activity in mouse lungs and macrophages. C57B/6 mice were infected with different doses of PR8 or saline by intranasal inoculation, and the lung tissue was collected on day 3 after infection. The PPARγ activity **(A)** and mRNA **(B)** in the mouse lung tissue were determined. The viral RNA in mice lungs was also determined by RT-PCR **(C)**. The isolated mouse lung alveolar epithelial type II cells (ATII **(D)**), alveolar macrophages (AM **(E)**), lung interstitial macrophages (IM **(F)**), and bone marrow-derived macrophages (BMDM **(G)**) were infected with PR8 at MOI=1, harvested the cell pellet at 24 h after infection for RNA extraction. The PPARγ mRNA was determined by RT-PCR. Unpaired t test was applied for the statistical analysis. *p < 0.05, **p < 0.01, ***p < 0.001 and ****p < 0.0001.

To investigate whether the influenza virus impairs PPARγ signaling *in vivo*, we investigated PPARγ in a mouse model of flu infection. We infected C57B/6 mice with different doses of PR8 by intranasal inoculation. Data in **Figure 4A** shows that PR8 infection decreased PPARγ activity dose-dependently. **Figures 4B**, **C** shows that viral infection significantly decreased PPARγ mRNA. To verify that influenza virus infection decreases the PPARγ activity in mouse macrophages, we isolated other macrophages, including alveolar macrophage, lung intestinal macrophage, bone marrow macrophage, and type II alveolar epithelial cell (ATII) from the mouse and then infected these cells with PR8. 24 hpi after PR8 infection, determined the mRNA level of PPARγ. Our results suggested that PR8 only decreased PPARγ activity in mouse macrophages but not in ATII, similar to our observation in primary human cells (**Figures 4D–G**).

### Activation of PPARγ ameliorated influenza-induced lung injury and improved survival

**Figure 5:**
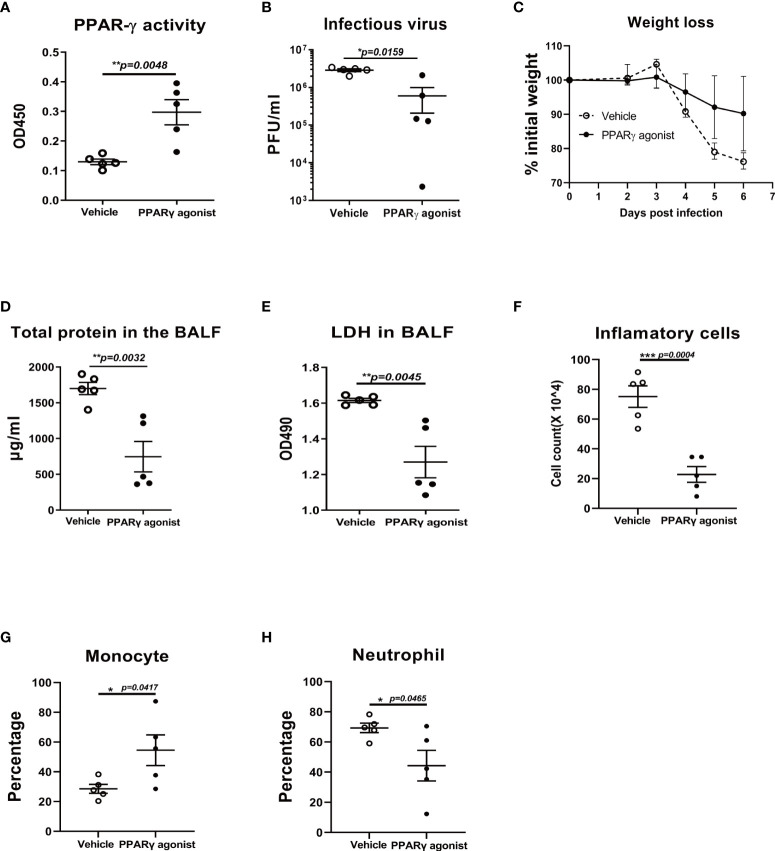
PPARγ agonist reduces viral replication and virus-induced weight loss in influenza virus-infected mice. C57B/6 mice were treated with PPARγ agonist troglitazone prior to influenza infection and evaluated viral burden, weight loss, and inflammatory cell differentiation in bronchoalveolar lavage fluid (BALF) and acute lung injury. Compared to the vehicle control-treated group, troglitazone-treated mice displayed elevated PPARγ activity **(A)**, reduced viral burden **(B)** and attenuated weight loss **(C)**. There was a significantly decreased total protein **(D)** and LDH **(E)**, reduced total cell number **(F)**, reduced percentage of neutrophils **(G)**, and an increased percentage of monocytes in the infected BALF **(H)**. Unpaired t test was applied for statistical analysis. *p < 0.05, **p < 0.01, and ***p < 0.001.

To further determine whether the acute lung injury is due to an impaired PPARγ pathway. We treated mice with PPARγ agonist troglitazone prior to influenza infection and evaluated viral burden, weight loss, inflammatory cell differentiation in bronchoalveolar lavage fluid (BALF), and acute lung injury. Compared to the vehicle control-treated group, troglitazone-treated mice displayed elevated PPARγ activity (**Figure 5A**), reduced viral burden (**Figure 5B**), and attenuated weight loss (**Figure 5C**). There was a significantly decreased total protein and LDH, total cell number, percentage of neutrophils, and an increased percentage of monocytes in the infected BAL (**Figures 5D-H**). These data indicate that virus-induced lung injury is dependent on the downregulation of the PPARγ pathway.

### PPARγ antagonist enhanced the inflammatory response and exacerbated damage in the lung of influenza-infected mice

**Figure 6:**
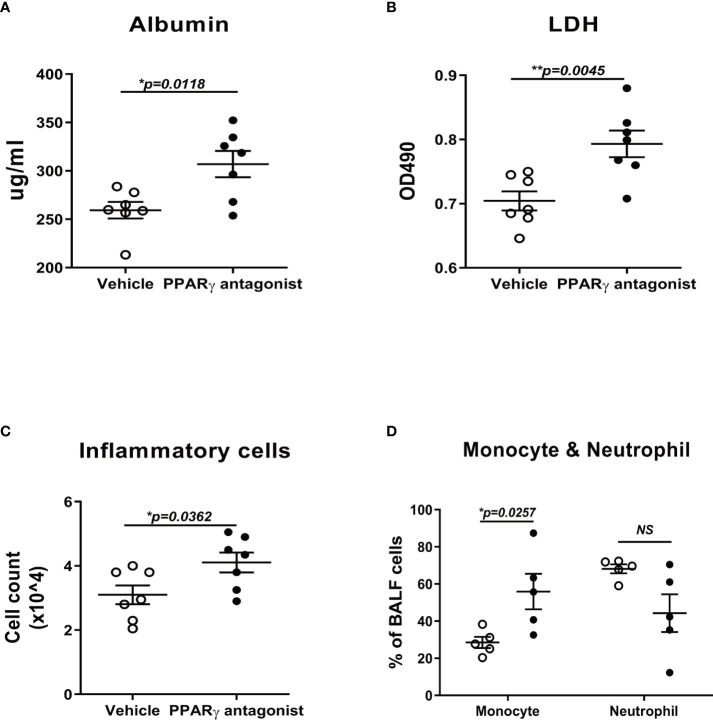
PPARγ antagonist promotes influenza-induced lung injury and inflammation. C57B/6 mice were treated with PPARγ antagonist, T0070907, prior to influenza infection and evaluated inflammatory cell differentiation in bronchoalveolar lavage fluid (BALF) and acute lung injury. Compared to the vehicle control-treated group, T0070907 treated mice displayed elevated albumin **(A)**, LDH **(B)**, TNFα **(C)**, and total cell number **(D)**. Unpaired t test was applied for the statistical analysis. ns means no significance. *p < 0.05, **p < 0.01.

Since PPARγ agonist ameliorated the lung injury during flu infection in mice, we investigated whether the PPARγ antagonist could worsen the lung injury or not. In the antagonist treatment group, albumin and LDH, the two important indicators of lung injury, were significantly elevated in antagonist-treated mice lungs (**Figures 6A**, **B**). In addition, the total inflammatory cell number and the portion of monocytes were also significantly elevated under the treatment of the antagonist (**Figures 6C**, **D**).

## Discussion

The influenza virus is a common public health problem and can cause serious complications, including events leading to hospitalization and death (21). The mortality due to influenza has been associated with excessive inflammatory response and cytokemia (22). Recently, several studies have highlighted the importance of therapies targeting host inflammatory responses. Alveolar macrophages are target cells in the lung infected by influenza virus and release many proinflammatory modulators during influenza infection to restrain viral infection (17, 23). PPARγ is expressed in monocytes and macrophages (24). In addition to its well-known regulatory effects on lipid and glucose metabolism, PPARγ is an important anti-inflammatory transcription factor that antagonizes NF-κB mediated cytokine production (25).

In this study, we have demonstrated that IAV infection decreased gene expression and transcript activity of PPARγ in human primary AMs, and there is a dose-dependent relationship between the PR8 infection and decreased PPARγ activity, as shown in **Figure 1**. Furtherly, knock-down of PPARγ by siPPARγ enhanced the secretion of multiple cytokines in primary human AMs (**Figures 2B-E**). On the other hand, activation of PPARγ in human AMs treated by PPARγ agonist reduced secretion of multiple cytokines. (**Figure 2G-J**). Also, PPARγ agonist treated mouse AMs showed less secretion of TNFα during IAV infection(**Figure 2K**). At the same time, the antagonist diminished the inflammatory inhibition effect of the PPARγ agonist during IAV infection (**Figures 2L**, **M**). Taken together, these results suggested that PPARγ plays an important role in the IAV infection-induced inflammatory response in human AMs. Although a reduction of multiple inflammatory factors was observed in PPARγ-activated AM, the expression of IFN was not significantly reduced (**Figure 3**). We got the similar resuts in H3N2 infected human primary AMs (**Supplementary Figure 1**).

In addition to investigate the role of PPARγ during IAV infection in human primary AMs, we infected mice with IAV and harvested the lungs to investigate the PPARγ activity and mRNA level. We found that the IAV infection decreased the PPARγ activity and mRNA level (**Figures 4A–C**) in mouse lung. In order to verify the IAV infection inhibited the expressing level of PPARγ in what kind cells, ATII cells and different macrophages, including alveolar macrophages, interstitial macrophages, and bone-marrow-derived macrophages were isolated from the naive mice and cultured for IAV infection. Our results suggested that IAV infection only inhibited the PPARγ expressing in macropahges but not in the ATII cells (**Figures 4D–G**).

Since the IAV infection significantly reduced the PPARγ activity in mouse lung, we hypothesized that activated the PPARγ activity *in vivo* moderate inflammatory response and immunopathological damage caused by IAV. As expected, PPARγ activation protected against IAV-induced lung injury and mortality in mice (**Figure 5**). Conversely, PPARγ antagonist treated mice displayed worsened mortality and delayed viral clearance (**Figure 6**).

Cytokine storm, the consequence of misregulation of inflammatory cytokines and hyperactivation of the innate immune response, has been recognized as a key mediator of influenza-induced lung disease and may be the key to COVID-19 infection (15, 26). Stimulation of PPARγ by natural or synthetic agonists may modulate the cytokine storm typical of viral infection by preventing cytokine overproduction and the inflammatory cascade (6) (27). Several natural PPARγ ligands have been proposed to treat COVID-19 (8). Due to its ability to reduce inflammatory parameters, the PPARγ agonist pioglitazone has also been proposed as an effective treatment for COVID-19 patients with diabetes, hypertension, and cardiovascular comorbidities (28).

There are several recent studies reported that treatment with PPARγ agonists significantly reduced flu-associated pathogenesis. Our results are consistent with the results from Jie Sun’s group that PPARγ deficiency enhaces mouse susceptibility to influenza-induced mortality (12).

Gopal et al. did not reveal that PPARγ plays a key anti-inflammatory role in what kind of cells (29). To our knowledge, there is no published paper using human primary macrophages to investigate the role of PPARγ during IAV infection. In the present study, we tested and verified the decreased PPARγ activity in human primary lung macrophages and different mouse macrophages including AM, IM, and BMDM during influenza virus infection. Our results revealed that PPARγ mainly exerts anti-inflammatory effects in human macrophages during IAV infection which had not been reported in any published paper. Some other published papers also investigated the anti-inflammatory effects of PPARγ, but these studies focused on different pathogens and the role of PPARγ in different kind cells like neutrophil but not in the macrophages (30–32). Our study used *in vitro* human primary cell culture and an *in vivo* mouse model of IAV infections, whereas the other focused solely on a mouse model.

Our results have shown that flu infection directly targets macrophages to reduce PPARγ activity and expression, causing lung injury and inflammation. In addition, a study with the respiratory syncytial virus (RSV) has shown a down-regulation of PPARγ expression by a nonstructural protein of RSV virus, and PPARγ agonists have beneficial effects in the suppression of the inflammatory response during RSV infection and therefore might have clinical efficacy in the course of severe RSV-infection (33). To this point, our study provides the mechanistic rationale for anti-inflammatory therapy through PPARγ for influenza and maybe other respiratory infections, including RSV and SARS- Cov2. Further studies to evaluate the effect of the various PPARγ agonists against viral infection will be worthwhile.

The current study provides a novel mechanism by which the influenza virus destroys the anti-inflammatory balance in the lung and causes acute lung injury. In conclusion, we performed *in vitro* and *in vivo* studies to determine the role of PPARγ in influenza infection. This study demonstrates that IAV reduces the transcriptional activity of PPARγ, which is critical for influenza-induced acute lung injury and mortality. Our findings suggest that PPARγ agonists have the potential to be used to limit influenza-related mortality and morbidity.

## Data availability statement

The original contributions presented in the study are included in the article/**Supplementary Material**. Further inquiries can be directed to the corresponding authors.

## Ethics statement

The animal study was reviewed and approved by The University of Pittsburgh Institutional Animal Care and Use Committee. Human AMs were isolated from de-identified donors.

## Author contributions

HZ performed in vitro and in vivo experiments, collected data, and wrote the manuscript. TA performed PPARg agonist and antagonist experiments in human AMs. SL participated in vivo experiments. DZ edited the manuscript. JW designed experiments, summarized the data, and edited the manuscript. All authors contributed to the article and approved the submission.

## Funding

This work was supported by National Institutes of Health grants R03AI101953 (to J.W.), R01HL113655 (to J.W.), and startup funding from the University of Pittsburgh (to J.W.).

## Conflict of interest

The authors declare that the research was conducted in the absence of any commercial or financial relationships that could be construed as a potential conflict of interest.

## Publisher’s note

All claims expressed in this article are solely those of the authors and do not necessarily represent those of their affiliated organizations, or those of the publisher, the editors and the reviewers. Any product that may be evaluated in this article, or claim that may be made by its manufacturer, is not guaranteed or endorsed by the publisher.

## Supplementary material

Supplementary Figure 1 PPARγ agonist inhibits H3N2 induced secretion of IL-8 and TNFα but not IP-10. Isolated human AMs were treated with PPARγ agonist, troglitazone (5 μM) in human Ams after H3N2 virus infection. After infection, cells were cultured with troglitazone for another 24 h. At 24 h after infection, cell-free culture supernatants were collected to detect cytokines using DuoSet ELISA kits from R&D Systems. **(A)** IL-8. **(B)** TNFα. **(C)** IP-10. Unpaired t test was applied for the statistical analysis.γ
