# Supplementary figures and images for: Influenza virus causes lung immunopathology through down-regulating PPARγ activity in macrophages
Source: Front Immunol. 2022 Aug 25;13:958801. doi: 10.3389/fimmu.2022.958801 (PMC9452838; doi:10.3389/fimmu.2022.958801)

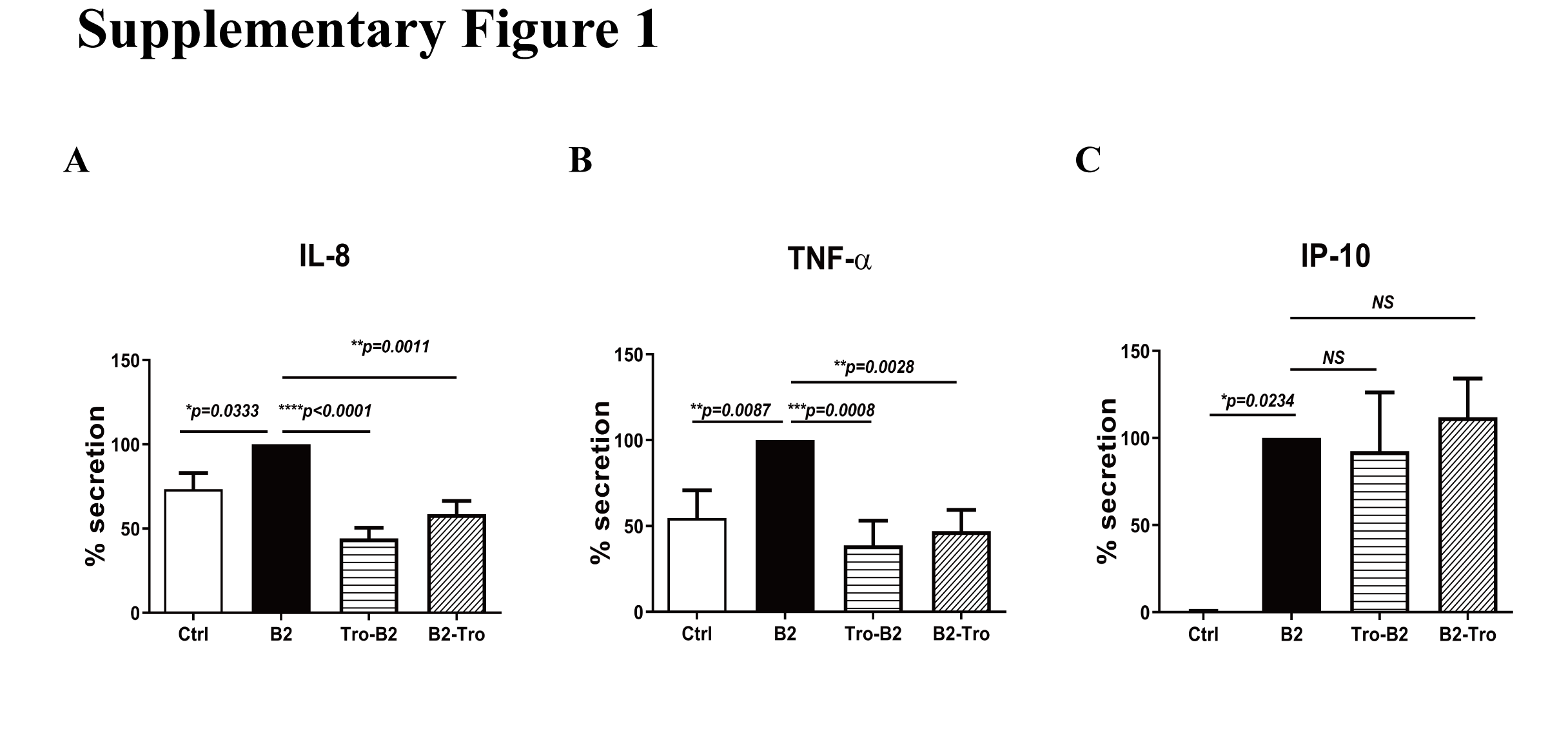

Supplement: Supplementary Figure 1 — PPARγ agonist inhibits H3N2 induced secretion of IL-8 and TNFα but not IP-10. Isolated human AMs were treated with PPARγ agonist, troglitazone (5 μM) in human Ams after H3N2 virus infection. After infection, cells were cultured with troglitazone for another 24 h. At 24 h after infection, cell-free culture supernatants were collected to detect cytokines using DuoSet ELISA kits from R&D Systems. (A) IL-8. (B) TNFα. (C) IP-10. Unpaired t test was applied for the statistical analysis.γ [file Image_1.tif]
